# Stepwise acquisition of unique epigenetic signatures during differentiation of tissue Treg cells

**DOI:** 10.3389/fimmu.2022.1082055

**Published:** 2022-12-08

**Authors:** Kathrin L. Braband, Tamara Kaufmann, Stefan Floess, Mangge Zou, Jochen Huehn, Michael Delacher

**Affiliations:** ^1^ Institute for Immunology, University Medical Center Mainz, Mainz, Germany; ^2^ Research Center for Immunotherapy, University Medical Center Mainz, Mainz, Germany; ^3^ Department of Experimental Immunology, Helmholtz Centre for Infection Research, Braunschweig, Germany; ^4^ Hannover Medical School, Hannover, Germany

**Keywords:** Treg, tissue, epigenetics, methylation, homeostasis

## Abstract

Regulatory T cells in non-lymphoid tissues are not only critical for maintaining self-tolerance, but are also important for promoting organ homeostasis and tissue repair. It is proposed that the generation of tissue Treg cells is a stepwise, multi-site process, accompanied by extensive epigenome remodeling, finally leading to the acquisition of unique tissue-specific epigenetic signatures. This process is initiated in the thymus, where Treg cells acquire core phenotypic and functional properties, followed by a priming step in secondary lymphoid organs that permits Treg cells to exit the lymphoid organs and seed into non-lymphoid tissues. There, a final specialization process takes place in response to unique microenvironmental cues in the respective tissue. In this review, we will summarize recent findings on this multi-site tissue Treg cell differentiation and highlight the importance of epigenetic remodeling during these stepwise events.

## Treg cells prevent autoimmunity and promote tissue regeneration

Regulatory T (Treg) cells are an anti-inflammatory, immunoregulatory subtype of CD4^+^ T cells and are of crucial importance for the maintenance of self-tolerance. At the molecular level, this cell type is characterized by the expression of Foxp3, which endows these immunoregulatory cells with suppressive activity (1, 2). Accordingly, mutations or deletion of *Foxp3* can cause catastrophic autoimmune events leading to the so-called “Scurfy” phenotype in mice or the IPEX (immune dysregulation, polyendocrinopathy, enteropathy, X-linked) syndrome in humans (3–6). Although stable Foxp3 expression is of utmost importance for lineage identity and functionality of Treg cells (7–9), mere Foxp3 expression is not sufficient to ensure complete Treg cell phenotypic and functional properties (10, 11). Instead, accumulating evidence suggests that in addition to Foxp3 expression the DNA demethylation at a set of Treg cell-specific epigenetic signature genes is necessary for complete Treg cell functionality and long-term lineage stability (8, 12–14).

**Figure 1:**
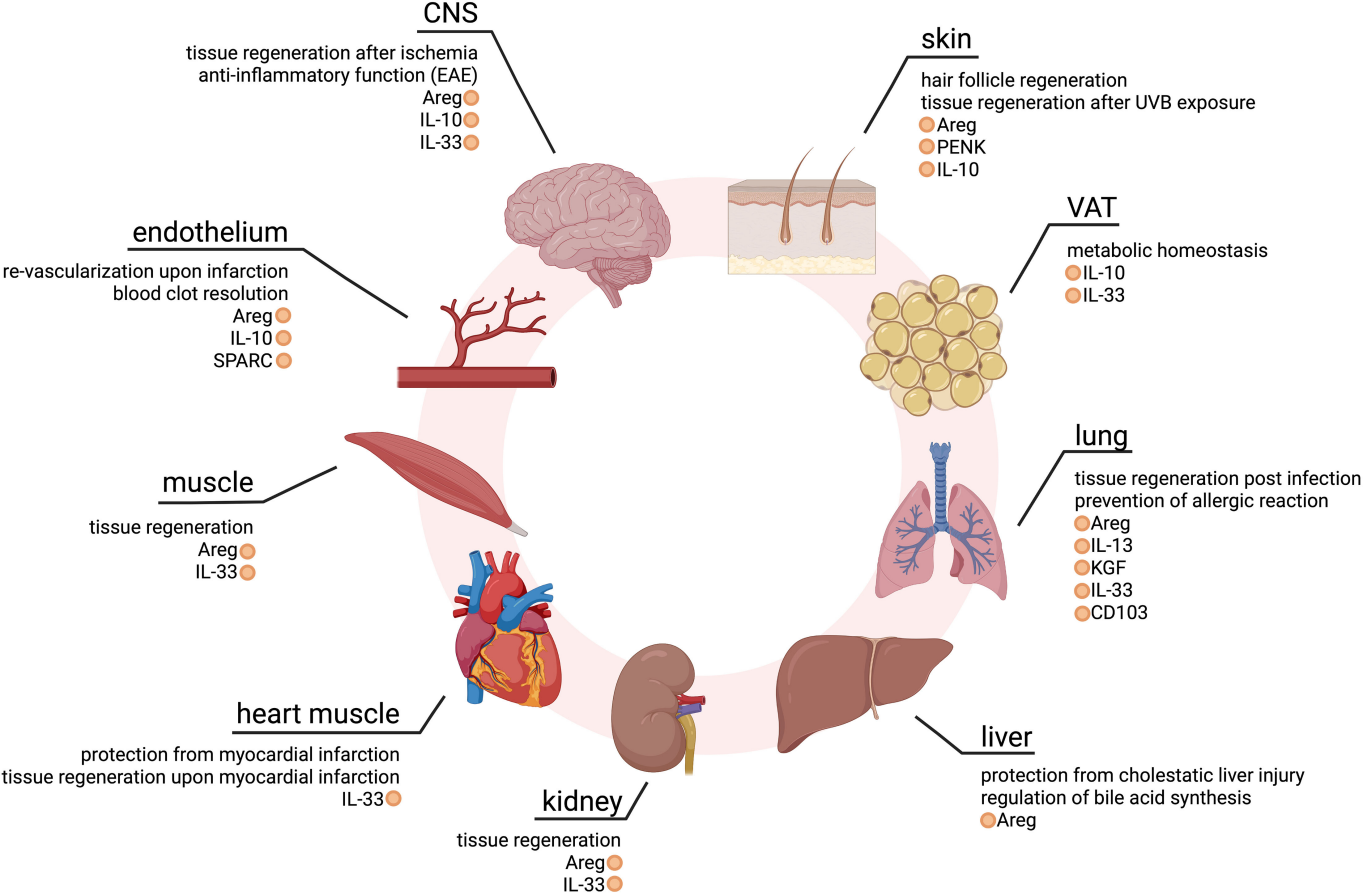
Overview of non-classical Treg cell functions in different tissues. Effector molecules mediating homeostatic or tissue-regenerative functions are marked in orange. CNS, central nervous system; VAT, visceral adipose tissue. Created with BioRender.com.

Due to the fatal systemic consequences with autoimmune events and multi-organ immune invasion and destruction upon mutation or deletion of *Foxp3*, Treg cells are usually described as “guardians of peripheral immune tolerance”. In recent years, however, it has become evident that Treg cells can also be found in non-lymphoid tissues (NLTs), where they perform important homeostatic and regenerative functions (15, 16). One prime example of a non-classical function of tissue Treg cells is from the visceral adipose tissue (VAT): In the murine system, local and systemic metabolic homeostasis has been linked to the presence and proper function of VAT-resident Treg cells, dependent on the interleukin (IL)-33 - ST2 axis, and tissue Treg cells expressing ST2 have also been found in the human system (17–19). In murine skin tissue, Treg cells have been shown to home to hair follicles, thus promoting hair follicle regeneration (20), and human autoimmune hair loss has been linked to impaired Treg cell function (21, 22). Treg cells further play a major role in wound healing and skin barrier regeneration upon UVB damage, in an Amphiregulin (Areg)- and Proenkephalin (PENK)-dependent manner in mice (23–25). Another example is from the lung tissue, where a specific population of Treg cells resides *in situ* and promotes tissue repair in an IL-33-dependent fashion. This population is required for preventing severe acute lung damage in an influenza A virus (IAV) infection model (26, 27), and promotes tissue repair after acute lung injury in mice (28–30), supporting lung epithelial proliferation (31) and reducing fibroproliferation (32). This cell type is also found in humans with acute lung injury, and upon stimulation has been reported to express keratinocyte growth factor (KGF), analogous to the murine system (28, 29). Treg cell-specific deletion of *Areg*, encoding for a key effector molecule of murine tissue repair-promoting Treg cells, leads to severe lung damage and decreased blood oxygen concentration during IAV infection (33). Both murine and human tissue Treg cells have further been shown to suppress the response to environmental allergens in the lungs (34). In the murine liver, tissue-resident Treg cells play a homeostatic role, regulating bile acid synthesis and protecting from cholestatic liver injury, dependent on Areg (35). Also, in an experimental mouse model of crescentic glomerulonephritis, Treg-derived Areg mediates tissue repair (36). In contrast to the abovementioned tissues, Treg cells can hardly be detected in healthy murine muscle. Upon muscle damage, however, repair-type Treg cells rapidly accumulate and secrete Areg to boost satellite cell function (37). Selective removal of Treg cells during muscle healing impairs muscle repair, while an increased influx of Treg cells enhances this process. In addition, Treg cells can be found in the muscle of genetically dystrophic mice, probably as a means of the body to regenerate ongoing muscular damage (38, 39). Tissue-resident Treg cells have also been reported in the heart, where they promote tissue regeneration after myocardial infarction. In murine ischemia/reperfusion injury and cryoinjury models, Treg cell-derived secreted protein acidic and rich in cysteine (SPARC) was identified as an important factor to protect the heart against myocardial infarction (40). Resident Treg cells further improve regeneration after infarction by modulating monocyte/macrophage differentiation (41). In the central nervous system (CNS), the deletion of Treg cells increases brain damage and augments post-ischemic activation of inflammatory cells in a model of experimental brain ischemia. In this model, the immune modulatory cytokine IL-10 was shown to be key in preventing secondary infarct growth, and IL-10-deficient Treg cells were ineffective (42). Indeed, the accumulation of brain Treg cells potentiates neurological recovery during the chronic phase of ischemic brain injury (43, 44). Interestingly, they also secrete increased amounts of Areg to suppress neurotoxic astrogliosis (43). Another study investigated the importance of CNS-resident Treg cells in experimental autoimmune encephalomyelitis (EAE), an animal model of multiple sclerosis, and reported a rapid accumulation of Treg cells in the CNS (45). There, Treg cells promote myelin regeneration and oligodendrocyte differentiation, thus contributing to brain regeneration *in situ* (46). In addition, Treg cells can limit neuroinflammation in this model, in an ST2-dependent manner (47). The importance of Treg cells has further been shown in the context of post-ischemic re-vascularization in mice, promoting endothelial cell proliferation and function (48), and in blood clot resolution, dependent on SPARC (49). In summary, these studies demonstrate that Treg cells have, beyond the ability to regulate immune responses, a multitude of non-canonical features that promote tissue regeneration and repair in many different organs (**Figure 1**).

## Treg cell development in the thymus

It is assumed that the majority of the Treg cell population originates from the thymus, termed thymus-derived Treg (tTreg) cells (50). Similar to the clonal deletion of self-reactive thymocytes, the development of tTreg cells is driven by the intrathymic encounter of agonist self-antigens, and expression of tissue-restricted antigens (TRAs) in medullary thymic epithelial cells (mTECs) plays a critical role in this process (50). tTreg cell generation occurs in two distinct developmental steps, identified by different expression levels of CD25 and Foxp3. The first developmental step is instructed by concurrent T cell receptor (TCR) signaling and co-stimulation, whereas the second step was shown to depend on signals derived from common gamma chain (γ_c_) cytokines, particularly IL-2 (51–56). Although both CD25^+^Foxp3^-^ and CD25^-^Foxp3^lo^ Treg precursor cells contribute almost equally to the generation of mature tTreg cells, it was recently reported that they show distinct maturation kinetics and cytokine responsiveness, differ in their transcriptomes and TCR repertoire, and only Treg cells derived from CD25^+^Foxp3^-^ progenitors protect against experimental autoimmune encephalitis (57). Yet, whether CD25^+^Foxp3^-^ Treg precursor cells preferentially give rise to tissue-resident Treg cells, is still unknown.

Accumulating evidence suggests that the establishment of the core Treg cell-specific epigenetic signature, consisting of *Foxp3*, *Tnfrsf18*, *Ctla4*, *Ikfz4* and *Il2ra*, occurs during tTreg cell development and maturation in a dynamic and progressive manner (13, 14, 58, 59). The two Treg cell precursors display distinct dynamics for the imprinting of the Treg cell-specific epigenetic signature genes, with CD25^-^Foxp3^lo^ Treg precursor cells already showing a partially established Treg cell-specific hypomethylation pattern (58, 60). This strongly suggests that at least a part of the Treg cell-specific epigenetic signature is already engraved into developing tTreg cells before they have entered the very transient CD25^-^Foxp3^lo^ Treg precursor cell stage. This assumption is in line with the finding that this unique hypomethylation pattern can be fully established even in the absence of Foxp3 expression (13). Furthermore, IL-2 signals mainly impact the demethylation of *Foxp3* with a more pronounced effect on CD25^-^Foxp3^lo^ when compared to CD25^+^Foxp3^-^ Treg precursor cells (58, 60). Thus, the two thymic Treg cell precursors differ substantially in the establishment of the Treg cell-specific hypomethylation pattern.

The shaping of the Treg cell-specific epigenetic signature during tTreg cell development relies on the interaction of the developing tTreg cells with thymic antigen-presenting cells, including thymic dendritic cells (DCs) and medullary thymic epithelial cells (mTECs). These cells display unique properties and were reported to induce more pronounced demethylation of the Treg cell-specific epigenetic signature genes in developing Treg cells when compared to splenic DCs (61). Mechanistically, the imprinting of the epigenetic signature occurs through an active process involving enzymes of the Ten-eleven-translocation (Tet) family, which act by iterative oxidation of 5mC to 5hmC, finally resulting in the demethylation of the CpG motif (14, 60, 62). In this regard, IL-2 was shown to maintain Tet2 expression at high levels during tTreg development, thereby protecting the Treg-specific demethylated region (TSDR) within *Foxp3* from re-methylation and fostering stable Foxp3 expression (62, 63). Furthermore, IL-2 can also directly modulate the epigenetic landscape of tTreg cells by regulating the positioning of the pioneer factor Satb1 in CD4 single-positive (SP) thymocytes and controlling genome-wide chromatin accessibility (64). Satb1 is crucial for Treg cell super-enhancer establishment and thus for the induction of Treg cell signature genes, and developmental stage-specific deletion of Satb1 leads to autoimmunity due to Treg cell deficiency (65).

In addition to the newly generated tTreg cells, the thymus also harbors recirculating Treg cells entering the thymus from the periphery. It was shown that these recirculating Treg cells exert a regulatory function by competing for IL-2 and thereby limiting the generation of new tTreg cells under steady-state conditions (66). More recently, we could demonstrate that a subset of recirculating IL-1R2^+^ Treg cells can quench IL-1 signaling to maintain tTreg cell development even under inflammatory conditions (67). It is important to note that the population of recirculating Treg cells is composed of multiple subsets and displays the most diverse TCR repertoire of all CD4 SP thymocytes (68). Yet, to the best of our knowledge, it has not been studied whether recirculating Treg cells also contribute to a tissue-specific negative feedback mechanism controlling the development of designated tissue Treg precursor cells by competing for specific TRAs on mTECs.

## Tissue Treg precursor cells in secondary lymphoid organs

**Figure 2:**
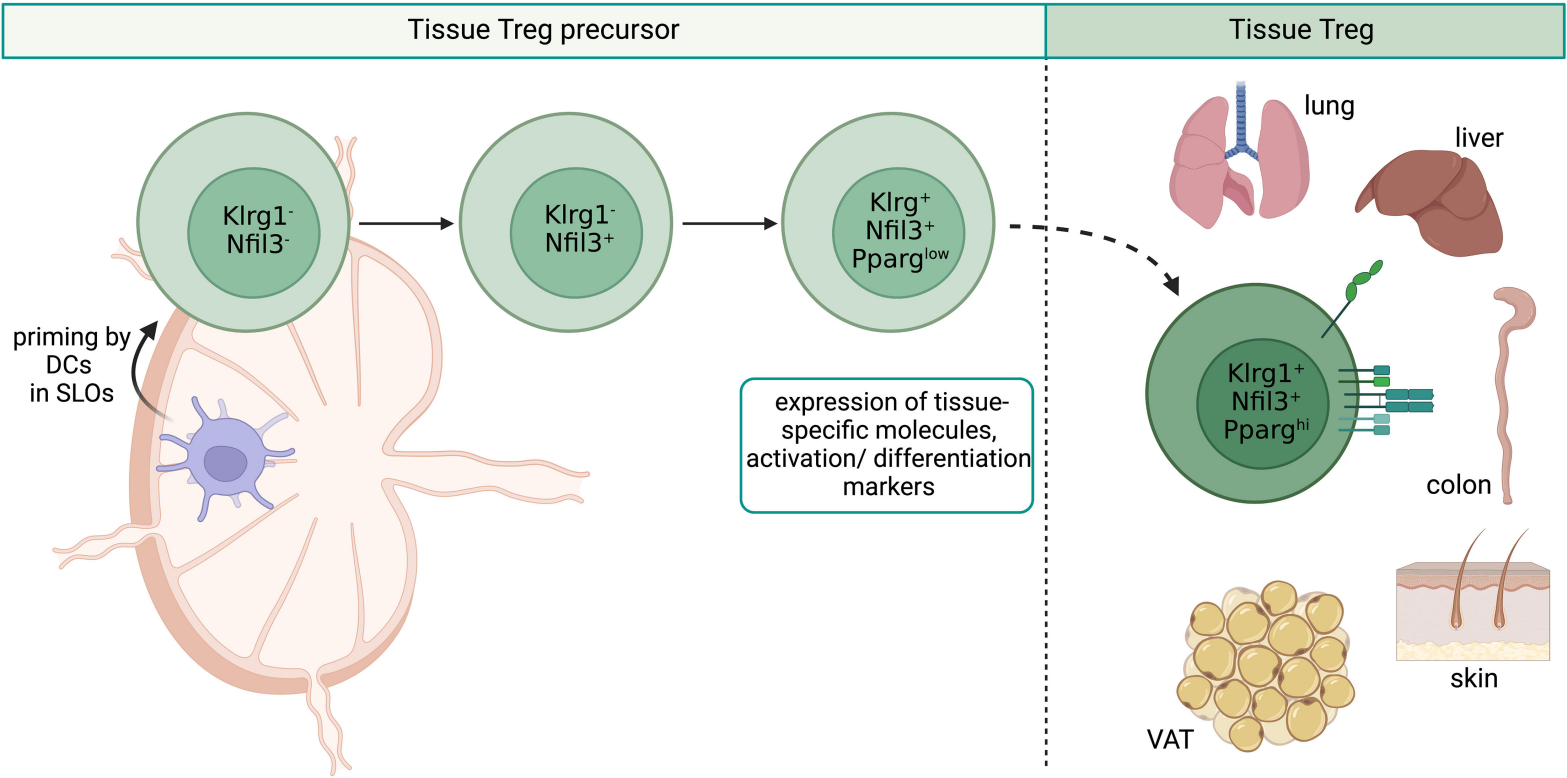
Overview of tissue Treg cell epigenetics in SLOs. Priming by DCs in SLOs leads to the stepwise development of tissue Treg cell precursors from Klrg1^-^Nfil3^-^ over Klrg1^-^Nfil3^+^ to Klrg1^+^Nfil3^+^. Klrg1^+^Nfil3^+^ Treg cell precursors additionally express low levels of PPARγ, tissue-specific genes like *Itgb1*, *Cxcr3*, *Ccr9*, *Itga4*, and activation/differentiation related genes such as *Klrg1*, *Pdcd1* and *Rora*. Klrg1^+^Nfil3^+^ Treg cell precursors migrate to NLTs like VAT, skin, lungs, liver and colon. SLOs, secondary lymphoid organs; DCs, dendritic cells; Treg, regulatory T cell; NLTs, non-lymphoid tissues; VAT, visceral adipose tissue. Created with BioRender.com.

The next step of tissue Treg cell generation, the priming in secondary lymphoid organs (SLOs), is less completely understood. We had previously demonstrated the existence of naive-like, recirculating and effector/memory-like, inflammation-seeking Treg cells in SLOs (69), and could show that Treg cells acquire the expression of tissue-specific homing molecules when getting primed by DCs in tissue-draining lymph nodes (70). First experimental proof that such priming events lead to the generation of tissue Treg cells came from a more recent study, in which a TCR-transgenic system was used to describe in detail the generation of the paradigmatic tissue Treg cell population, the VAT-Treg cells (71). Here, the authors could show that a part of the VAT-Treg cell signature was already induced in a fraction of splenic Treg cells marked by low expression levels of the transcription factor PPARγ (peroxisome proliferator-activated receptor gamma) and that these PPARγ^low^ Treg cells displayed a high VAT-seeding potential. In a follow-up study, the tissue Treg precursor cell concept could be further generalized by demonstrating that the splenic PPARγ^low^ Treg cell population is transcriptionally highly heterogeneous and engenders Treg cells in multiple NLTs beyond VAT, such as skin, colon and liver. These tissue Treg cell precursors express genes encoding for migration-related surface molecules such as *Itgb1*, *Cxcr3*, *Ccr9*, *Itga4* and activation/differentiation associated proteins such as *Klrg1*, *Pdcd1* and *Rora* (72, 73). These findings are in accordance with our own observations using a novel Nfil3 (nuclear factor, interleukin-3 regulated) reporter mouse line to track tissue Treg cell development (**Figure 2**). We could identify early (*Nfil3* ^GFP+^Klrg1^-^) and late (*Nfil3* ^GFP+^Klrg1^+^) tissue Treg precursor cells in SLOs, and global profiling studies revealed a stepwise acquisition of the mature tissue Treg cell phenotype (74). However, a detailed view of the DNA methylation profile of Treg cell precursors in SLOs is still missing, which could provide further insights into the epigenetic preparation of designated tissue Treg cells during their priming in the spleen and lymph nodes.

## Tissue Treg cells acquire unique epigenetic signatures within tissues

**Figure 3:**
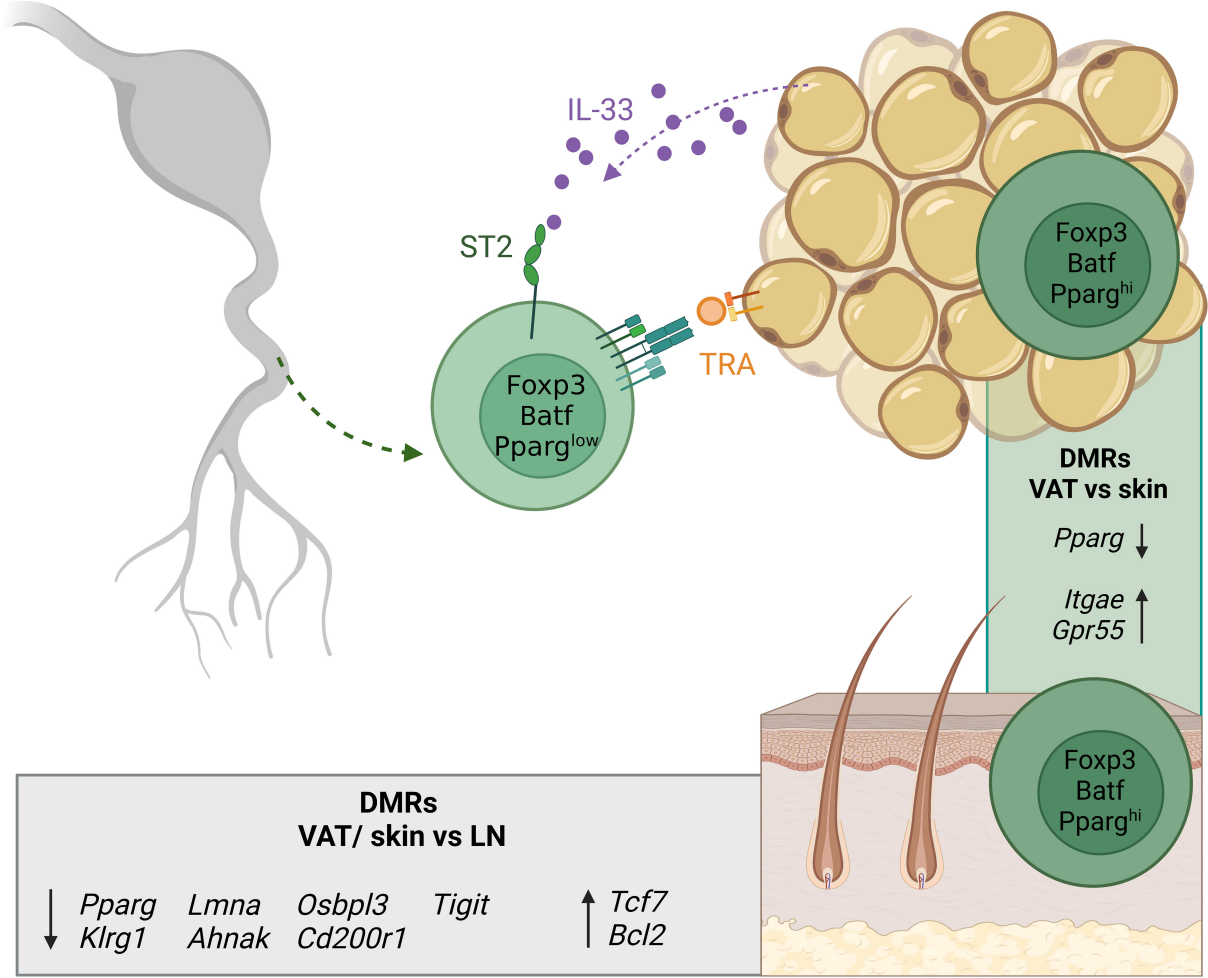
Overview of tissue Treg cell epigenetics in tissues. Tissue Treg precursors leave SLOs already expressing low levels of PPARγ and are recruited to the tissues (here exemplified by VAT) *via* soluble factors like IL-33 and *via* TRA recognition. In the tissue, the tissue Treg-specific methylation pattern is completed. The grey box shows DMRs of Treg cells from VAT and skin versus lymph node-derived Treg cells. The green box shows DMRs between VAT and skin Treg cells. Treg, regulatory T cell; SLOs, secondary lymphoid organs; VAT, visceral adipose tissue; LN, lymph node; TRA, tissue-restricted antigen; DMRs, differentially-methylated regions. Created with BioRender.com.

Recent single-cell profiling studies suggested that tissue Treg cells display tissue-specific signatures dependent on the unique tissue environment that are distinct from those of lymphoid organ Treg cells. A common core-signature could further be identified between mouse and human (73, 75). Analysis of the VAT suggests that the extravasation and accumulation of tissue Treg cells relies on the TCR-dependent recognition of TRAs and on unique cytokine signals (71). However, studies from other tissues are lacking so far. Tissue-specific functional adaptations come along with additional epigenetic alterations as demonstrated in a whole-genome bisulfite sequencing (WGBS) study of murine lymph node-derived Treg and conventional T (Tconv) cells, as well as fat- and skin-derived Treg cells (76). In this study, we could identify 339 differentially-methylated regions (DMRs) between Treg and Tconv cells, which constitute the abovementioned core Treg cell-specific epigenetic signature with *Ctla4*, *Il2ra*, *Il2rb*, *Ikzf2* and *Ikzf4*, and *Foxp3* as its most prominent members. Strikingly, about five times more DMRs describe the difference between Treg cells derived from lymph node and skin (1645 regions) and lymph node and fat (1593 regions), with *Pparg, Klrg1* and *Tigit* as well-known examples of hypomethylated regions, and *Bcl2* and *Tcf7* of hypermethylated regions in tissue- vs lymphoid organ Treg cells (**Figure 3**). Thus, the epigenetic signature of Treg cells in their tissue-specific microenvironment comprises severalfold more DMRs than the core Treg cell-specific epigenetic signature itself. Although tissue Treg cells have an activated phenotype, scATAC-sequencing of naïve and activated lymphoid Treg cells and also Treg cells from murine skin, VAT and colon revealed clear differences in the epigenetic profiles of activated lymphoid- and tissue Treg cells. This indicates a major impact of the tissue signature on the latter (74). In addition, we could define both tissue-specific signatures and a global ‘tissular’ epigenetic framework with a key role for the transcription factor Basic leucine zipper transcription factor, ATF-like (Batf). The correlation between gene expression and negative mean methylation values confirms the dogma of epigenetics: specific demethylation in the gene body corresponds to the expression of the associated gene, and vice versa. In addition, we identified tissue-specific methylation patterns, such as demethylated regions in *Ahr*, *Icos*, *Itgae* and *Gpr55* in skin Treg cells. In this context, although it is widely believed that the definitive tissue Treg cell phenotype is not installed until the cells have settled down in the respective tissues and have progressively adapted to the tissue microenvironment, it is unknown so far which parts of the tissue-specific DNA methylation patterns are already induced in early (Nfil3^+^Klrg1^-^) and late (Nfil3^+^Klrg1^+^) tissue Treg precursor cells within SLOs, and how these programs influence the extravasation into the target tissue. Furthermore, although the impact of inflammatory conditions on the functional properties and epigenetic signature of tissue Treg cells has been intensively studied (77–79), the underlying molecular mechanisms are largely unknown. Further missing puzzle pieces will help to clarify whether the heterogeneous transcriptome of tissue Treg cells is also based on a heterogeneous methylome, and which molecular mechanisms are involved in modulating epigenetic signatures and functional properties in recently described inflammatory models.

The knowledge about the human Treg cell methylome is even more limited. To date, only few studies investigated the global CpG methylation landscape in human Treg and Tconv cell populations from peripheral blood (80, 81). Recently, we have generated single-cell chromatin accessibility profiles of human tissue Treg cells from healthy skin and subcutaneous fat, and identified a tissue-repair Treg cell signature with a prevailing footprint of the transcription factor BATF (82). This signature, combined with gene expression profiling and TCR fate mapping, allowed for the discovery of the human counterpart of murine tissue-repair Treg cells as a T follicular helper cell (T_FH_)-polarized CCR8^+^BATF^+^ subset of Treg cells, and also identified its circulating blood precursor cell in human blood (82). BATF has further been reported as an important transcription factor for Treg cell differentiation and activation not only in healthy tissue, but also in the tumor microenvironment (83). To which extend healthy tissue and tumor Treg cells share transcriptional and epigenetic programmes remains to be investigated in detail on a grand scale for different tissues and tumor entities.

## Outlook

The generation of tissue Treg cells is a stepwise, multi-site process, yet the precise dynamics and underlying molecular mechanisms contributing to the acquisition of the highly specialized epigenetic profiles and the unique functional properties of tissue Treg cells are only incompletely understood. Tissue Treg cells originate from the thymus, where they progressively develop into mature Treg cells. During this process, progressive demethylation of Treg-cell specific epigenetic signature genes takes place, and mature Treg cells leave the thymus with a specific DNA methylation pattern in combination with classical Treg cell properties mediated by actions of the Foxp3 transcription factor complex (84). In SLOs, Treg cells are primed by DCs, and tissue Treg precursor cells acquire the expression of tissue-specific homing molecules. They develop from Nfil3^-^Klrg1^-^ *via* Nfil3^+^Klrg1^-^ to Nfil3^+^Klrg1^+^ tissue Treg cell precursors, paralleled by the stepwise acquisition of a partial tissue-specific chromatin accessibility signature, potentially priming them to migrate from SLOs into NLTs. In the tissue, microenvironmental cues lead to the completion of the tissue Treg cell-specific epigenetic signature. Currently, it is still unknown at which stage the priming in the SLOs starts the “pre-tissue” program, which target genes are differentially methylated during this step, and how important these programs are for the designated tissue Treg cells to extravasate and survive in their cognate tissue. Furthermore, the exact timing of these events is unknown as it has been reported that the postnatal period is critical for the generation of the first wave of tissue Treg cells, which after seeding into tissues can stably persist until adulthood (85, 86).

Since most of our knowledge of tissue-resident Treg cells and their development comes from mouse studies, we can further explore the precise DNA methylation characteristics of tissue Treg cell development in SLOs in future studies. In a translational study, an analogous cell type to murine tissue Treg cells was recently proposed for the human system (82), and further investigations regarding the development and epigenetic programming are necessary to fully understand this cell type and also understand their future therapeutic potential. Owing to their special tissue-regenerative properties on top of classical Treg cell functions, tissue Treg cells make an attractive candidate for adoptive T cell transfer therapies in autoimmune settings like graft-versus-host disease (GvHD) or solid organ transplantation (87, 88). A major limitation here is the small number of tissue Treg cells that can be isolated from biopsies or even re-circulating tissue Treg cells from the peripheral blood of the patient. We have shown that a tissue-repair phenotype with a tissue Treg cell-specific DNA methylation pattern can be induced in human naïve Treg cells by T_FH_-polarizing cytokines (82). It might therefore be possible to isolate classical Treg cells from the peripheral blood of patients, expand them *in vitro*, and induce the tissue Treg-specific repair phenotype. This would allow for the adoptive transfer of sufficient numbers of autologous Treg cells that have the potential not only to dampen auto-immune destruction but also to mediate regeneration of the damaged tissue. However, it is not known whether inducing a tissue-like program is enough to also promote extravasation into the target tissues and to promote tissue homeostasis and even organ regeneration *in situ* while promoting immune tolerance to the graft, a highly desirable feature both in bone marrow transfusion and transplantation medicine.

## Author contributions

Conceptualization and design: MD and JH. Literature search and Manuscript writing: MD, JH, KLB, TK, SF, MZ. Figures: KLB and TK. Manuscript revision: KLB, MD, JH. All authors contributed to the article and approved the submitted version.

## References

[1] Fontenot JD Gavin MA Rudensky AY. Foxp3 programs the development and function of CD4+CD25+ regulatory T cells. Nat Immunol (2003) 4(4):330–6. doi: 10.1038/ni904 12612578

[2] Hori S Nomura T Sakaguchi S. Control of regulatory T cell development by the transcription factor Foxp3. Science (2003) 299(5609):1057–61. doi: 10.1126/science.1079490 12522256

[3] Brunkow ME Jeffery EW Hjerrild KA Paeper B Clark LB Yasayko SA. Disruption of a new forkhead/winged-helix protein, scurfin, results in the fatal lymphoproliferative disorder of the scurfy mouse. Nat Genet (2001) 27(1):68–73. doi: 10.1038/83784 11138001

[4] Wildin RS Ramsdell F Peake J Faravelli F Casanova JL Buist N. X-Linked neonatal diabetes mellitus, enteropathy and endocrinopathy syndrome is the human equivalent of mouse scurfy. Nat Genet (2001) 27(1):18–20. doi: 10.1038/83707 11137992

[5] Bennett CL Christie J Ramsdell F Brunkow ME Ferguson PJ Whitesell L. The immune dysregulation, polyendocrinopathy, enteropathy, X-linked syndrome (IPEX) is caused by mutations of FOXP3. Nat Genet (2001) 27(1):20–1. doi: 10.1038/83713 11137993

[6] Khattri R Cox T Yasayko SA Ramsdell F. An essential role for scurfin in CD4+CD25+ T regulatory cells. Nat Immunol (2003) 4(4):337–42. doi: 10.1038/ni909 12612581

[7] Williams LM Rudensky AY. Maintenance of the Foxp3-dependent developmental program in mature regulatory T cells requires continued expression of Foxp3. Nat Immunol (2007) 8(3):277–84. doi: 10.1038/ni1437 17220892

[8] Floess S Freyer J Siewert C Baron U Olek S Polansky J. Epigenetic control of the foxp3 locus in regulatory T cells. PloS Biol (2007) 5(2):e38. doi: 10.1371/journal.pbio.0050038 17298177 PMC1783672

[9] Huehn J Polansky JK Hamann A. Epigenetic control of FOXP3 expression: the key to a stable regulatory T-cell lineage? Nat Rev Immunol (2009) 9(2):83–9. doi: 10.1038/nri2474 19114986

[10] Sugimoto N Oida T Hirota K Nakamura K Nomura T Uchiyama T. Foxp3-dependent and -independent molecules specific for CD25+CD4+ natural regulatory T cells revealed by DNA microarray analysis. Int Immunol (2006) 18(8):1197–209. doi: 10.1093/intimm/dxl060 16772372

[11] Hill JA Feuerer M Tash K Haxhinasto S Perez J Melamed R. Foxp3 transcription-factor-dependent and -independent regulation of the regulatory T cell transcriptional signature. Immunity (2007) 27(5):786–800. doi: 10.1016/j.immuni.2007.09.010 18024188

[12] Kim HP Leonard WJ. CREB/ATF-dependent T cell receptor-induced FoxP3 gene expression: A role for DNA methylation. J Exp Med (2007) 204(7):1543–51. doi: 10.1084/jem.20070109 PMC2118651 17591856

[13] Ohkura N Hamaguchi M Morikawa H Sugimura K Tanaka A Ito Y. T Cell receptor stimulation-induced epigenetic changes and Foxp3 expression are independent and complementary events required for treg cell development. Immunity (2012) 37(5):785–99. doi: 10.1016/j.immuni.2012.09.010 23123060

[14] Toker A Engelbert D Garg G Polansky JK Floess S Miyao T. Active demethylation of the Foxp3 locus leads to the generation of stable regulatory T cells within the thymus. J Immunol (2013) 190(7):3180–8. doi: 10.4049/jimmunol.1203473 23420886

[15] Panduro M Benoist C Mathis D. Tissue tregs. Annu Rev Immunol (2016) 34:609–33. doi: 10.1146/annurev-immunol-032712-095948 PMC4942112 27168246

[16] Malko D Elmzzahi T Beyer M. Implications of regulatory T cells in non-lymphoid tissue physiology and pathophysiology. Front Immunol (2022) 13:954798. doi: 10.3389/fimmu.2022.954798 35936011 PMC9354719

[17] Cipolletta D Feuerer M Li A Kamei N Lee J Shoelson SE. PPAR-gamma is a major driver of the accumulation and phenotype of adipose tissue treg cells. Nature (2012) 486(7404):549–53. doi: 10.1038/nature11132 PMC3387339 22722857

[18] Feuerer M Herrero L Cipolletta D Naaz A Wong J Nayer A. Lean, but not obese, fat is enriched for a unique population of regulatory T cells that affect metabolic parameters. Nat Med (2009) 15(8):930–9. doi: 10.1038/nm.2002 PMC3115752 19633656

[19] Vasanthakumar A Moro K Xin A Liao Y Gloury R Kawamoto S. The transcriptional regulators IRF4, BATF and IL-33 orchestrate development and maintenance of adipose tissue-resident regulatory T cells. Nat Immunol (2015) 16(3):276–85. doi: 10.1038/ni.3085 25599561

[20] Ali N Zirak B Rodriguez RS Pauli ML Truong HA Lai K. Regulatory T cells in skin facilitate epithelial stem cell differentiation. Cell (2017) 169(6):1119–29.e11. doi: 10.1016/j.cell.2017.05.002 28552347 PMC5504703

[21] Petukhova L Duvic M Hordinsky M Norris D Price V Shimomura Y. Genome-wide association study in alopecia areata implicates both innate and adaptive immunity. Nature (2010) 466(7302):113–7. doi: 10.1038/nature09114 PMC2921172 20596022

[22] Shin BS Furuhashi T Nakamura M Torii K Morita A. Impaired inhibitory function of circulating CD4+CD25+ regulatory T cells in alopecia areata. J Dermatol Sci (2013) 70(2):141–3. doi: 10.1016/j.jdermsci.2013.01.006 23433552

[23] Mathur AN Zirak B Boothby IC Tan M Cohen JN Mauro TM. Treg-cell control of a CXCL5-IL-17 inflammatory axis promotes hair-Follicle-Stem-Cell differentiation during skin-barrier repair. Immunity (2019) 50(3):655–67.e4. doi: 10.1016/j.immuni.2019.02.013 30893588 PMC6507428

[24] Nosbaum A Prevel N Truong HA Mehta P Ettinger M Scharschmidt TC. Cutting edge: Regulatory T cells facilitate cutaneous wound healing. J Immunol (2016) 196(5):2010–4. doi: 10.4049/jimmunol.1502139 PMC4761457 26826250

[25] Shime H Odanaka M Tsuiji M Matoba T Imai M Yasumizu Y. Proenkephalin(+) regulatory T cells expanded by ultraviolet b exposure maintain skin homeostasis with a healing function. Proc Natl Acad Sci U.S.A. (2020) 117(34):20696–705. doi: 10.1073/pnas.2000372117 PMC7456133 32769209

[26] Tan W Zhang B Liu X Zhang C Liu J Miao Q. Interleukin-33-Dependent accumulation of regulatory T cells mediates pulmonary epithelial regeneration during acute respiratory distress syndrome. Front Immunol (2021) 12:653803. doi: 10.3389/fimmu.2021.653803 33936076 PMC8082076

[27] Guo XJ Dash P Crawford JC Allen EK Zamora AE Boyd DF. Lung gammadelta T cells mediate protective responses during neonatal influenza infection that are associated with type 2 immunity. Immunity (2018) 49(3):531–44.e6. doi: 10.1016/j.immuni.2018.07.011 30170813 PMC6345262

[28] Dial CF Tune MK Doerschuk CM Mock JR. Foxp3(+) regulatory T cell expression of keratinocyte growth factor enhances lung epithelial proliferation. Am J Respir Cell Mol Biol (2017) 57(2):162–73. doi: 10.1165/rcmb.2017-0019OC PMC5576587 28296468

[29] D'Alessio FR Tsushima K Aggarwal NR West EE Willett MH Britos MF. CD4+CD25+Foxp3+ tregs resolve experimental lung injury in mice and are present in humans with acute lung injury. J Clin Invest (2009) 119(10):2898–913. doi: 10.1172/JCI36498 PMC2752062 19770521

[30] Liu Q Dwyer GK Zhao Y Li H Mathews LR Chakka AB. IL-33-mediated IL-13 secretion by ST2+ tregs controls inflammation after lung injury. JCI Insight (2019) 4(6):e123919. doi: 10.1172/jci.insight.123919 30779711 PMC6482994

[31] Mock JR Garibaldi BT Aggarwal NR Jenkins J Limjunyawong N Singer BD. Foxp3+ regulatory T cells promote lung epithelial proliferation. Mucosal Immunol (2014) 7(6):1440–51. doi: 10.1038/mi.2014.33 PMC4205163 24850425

[32] Garibaldi BT D'Alessio FR Mock JR Files DC Chau E Eto Y. Regulatory T cells reduce acute lung injury fibroproliferation by decreasing fibrocyte recruitment. Am J Respir Cell Mol Biol (2013) 48(1):35–43. doi: 10.1165/rcmb.2012-0198OC 23002097 PMC3547087

[33] Arpaia N Green JA Moltedo B Arvey A Hemmers S Yuan S. A distinct function of regulatory T cells in tissue protection. Cell (2015) 162(5):1078–89. doi: 10.1016/j.cell.2015.08.021 PMC4603556 26317471

[34] Faustino LD Griffith JW Rahimi RA Nepal K Hamilos DL Cho JL. Interleukin-33 activates regulatory T cells to suppress innate gammadelta T cell responses in the lung. Nat Immunol (2020) 21(11):1371–83. doi: 10.1038/s41590-020-0785-3 PMC7578082 32989331

[35] Santamaria E Rodriguez-Ortigosa CM Uriarte I Latasa MU Urtasun R Alvarez-Sola G. The epidermal growth factor receptor ligand amphiregulin protects from cholestatic liver injury and regulates bile acids synthesis. Hepatology (2019) 69(4):1632–47. doi: 10.1002/hep.30348 30411380

[36] Sakai R Ito M Komai K Iizuka-Koga M Matsuo K Nakayama T. Kidney GATA3(+) regulatory T cells play roles in the convalescence stage after antibody-mediated renal injury. Cell Mol Immunol (2021) 18(5):1249–61. doi: 10.1038/s41423-020-00547-x PMC8093306 32917984

[37] Burzyn D Kuswanto W Kolodin D Shadrach JL Cerletti M Jang Y. A special population of regulatory T cells potentiates muscle repair. Cell (2013) 155(6):1282–95. doi: 10.1016/j.cell.2013.10.054 PMC3894749 24315098

[38] Castiglioni A Corna G Rigamonti E Basso V Vezzoli M Monno A. FOXP3+ T cells recruited to sites of sterile skeletal muscle injury regulate the fate of satellite cells and guide effective tissue regeneration. PloS One (2015) 10(6):e0128094. doi: 10.1371/journal.pone.0128094 26039259 PMC4454513

[39] Kuswanto W Burzyn D Panduro M Wang KK Jang YC Wagers AJ. Poor repair of skeletal muscle in aging mice reflects a defect in local, interleukin-33-Dependent accumulation of regulatory T cells. Immunity (2016) 44(2):355–67. doi: 10.1016/j.immuni.2016.01.009 PMC4764071 26872699

[40] Xia N Lu Y Gu M Li N Liu M Jiao J. A unique population of regulatory T cells in heart potentiates cardiac protection from myocardial infarction. Circulation (2020) 142(20):1956–73. doi: 10.1161/CIRCULATIONAHA.120.046789 32985264

[41] Weirather J Hofmann UD Beyersdorf N Ramos GC Vogel B Frey A. Foxp3+ CD4+ T cells improve healing after myocardial infarction by modulating monocyte/macrophage differentiation. Circ Res (2014) 115(1):55–67. doi: 10.1161/CIRCRESAHA.115.303895 24786398

[42] Liesz A Suri-Payer E Veltkamp C Doerr H Sommer C Rivest S. Regulatory T cells are key cerebroprotective immunomodulators in acute experimental stroke. Nat Med (2009) 15(2):192–9. doi: 10.1038/nm.1927 19169263

[43] Ito M Komai K Mise-Omata S Iizuka-Koga M Noguchi Y Kondo T. Brain regulatory T cells suppress astrogliosis and potentiate neurological recovery. Nature (2019) 565(7738):246–50. doi: 10.1038/s41586-018-0824-5 30602786

[44] Gadani SP Walsh JT Smirnov I Zheng J Kipnis J. The glia-derived alarmin IL-33 orchestrates the immune response and promotes recovery following CNS injury. Neuron (2015) 85(4):703–9. doi: 10.1016/j.neuron.2015.01.013 PMC12064979 25661185

[45] Korn T Reddy J Gao W Bettelli E Awasthi A Petersen TR. Myelin-specific regulatory T cells accumulate in the CNS but fail to control autoimmune inflammation. Nat Med (2007) 13(4):423–31. doi: 10.1038/nm1564 PMC3427780 17384649

[46] Dombrowski Y O'Hagan T Dittmer M Penalva R Mayoral SR Bankhead P. Regulatory T cells promote myelin regeneration in the central nervous system. Nat Neurosci (2017) 20(5):674–80. doi: 10.1038/nn.4528 PMC5409501 28288125

[47] Hemmers S Schizas M Rudensky AY. T Reg cell-intrinsic requirements for ST2 signaling in health and neuroinflammation. J Exp Med (2021) 218(2): e20201234. doi: 10.1084/jem.20201234 33095261 PMC7590508

[48] Leung OM Li J Li X Chan VW Yang KY Ku M. Regulatory T cells promote apelin-mediated sprouting angiogenesis in type 2 diabetes. Cell Rep (2018) 24(6):1610–26. doi: 10.1016/j.celrep.2018.07.019 30089270

[49] Shahneh F Grill A Klein M Frauhammer F Bopp T Schafer K. Specialized regulatory T cells control venous blood clot resolution through SPARC. Blood (2021) 137(11):1517–26. doi: 10.1182/blood.2020005407 32932520

[50] Klein L Robey EA Hsieh CS. Central CD4(+) T cell tolerance: deletion versus regulatory T cell differentiation. Nat Rev Immunol (2019) 19(1):7–18. doi: 10.1038/s41577-018-0083-6 30420705

[51] Burchill MA Yang J Vang KB Moon JJ Chu HH Lio CW. Linked T cell receptor and cytokine signaling govern the development of the regulatory T cell repertoire. Immunity (2008) 28(1):112–21. doi: 10.1016/j.immuni.2007.11.022 PMC2430111 18199418

[52] Lio CW Hsieh CS. A two-step process for thymic regulatory T cell development. Immunity (2008) 28(1):100–11. doi: 10.1016/j.immuni.2007.11.021 PMC2248212 18199417

[53] Schuster M Glauben R Plaza-Sirvent C Schreiber L Annemann M Floess S. IkappaB(NS) protein mediates regulatory T cell development *via* induction of the Foxp3 transcription factor. Immunity (2012) 37(6):998–1008. doi: 10.1016/j.immuni.2012.08.023 23200824

[54] Tai X Erman B Alag A Mu J Kimura M Katz G. Foxp3 transcription factor is proapoptotic and lethal to developing regulatory T cells unless counterbalanced by cytokine survival signals. Immunity (2013) 38(6):1116–28. doi: 10.1016/j.immuni.2013.02.022 PMC3700677 23746651

[55] Schuster M Plaza-Sirvent C Visekruna A Huehn J Schmitz I. Generation of Foxp3(+)CD25(-) regulatory T-cell precursors requires c-rel and IkappaBNS. Front Immunol (2019) 10:1583. doi: 10.3389/fimmu.2019.01583 31354726 PMC6635800

[56] Apert C Galindo-Albarran AO Castan S Detraves C Michaud H McJannett N. IL-2 and IL-15 drive intrathymic development of distinct periphery-seeding CD4(+)Foxp3(+) regulatory T lymphocytes. Front Immunol (2022) 13:965303. doi: 10.3389/fimmu.2022.965303 36159793 PMC9495261

[57] Owen DL Mahmud SA Sjaastad LE Williams JB Spanier JA Simeonov DR. Thymic regulatory T cells arise *via* two distinct developmental programs. Nat Immunol (2019) 20(2):195–205. doi: 10.1038/s41590-018-0289-6 30643267 PMC6650268

[58] Herppich S Toker A Pietzsch B Kitagawa Y Ohkura N Miyao T. Dynamic imprinting of the treg cell-specific epigenetic signature in developing thymic regulatory T cells. Front Immunol (2019) 10:2382. doi: 10.3389/fimmu.2019.02382 31681278 PMC6797672

[59] Vanhanen R Leskinen K Mattila IP Saavalainen P Arstila TP. Epigenetic and transcriptional analysis supports human regulatory T cell commitment at the CD4+CD8+ thymocyte stage. Cell Immunol (2020) 347:104026. doi: 10.1016/j.cellimm.2019.104026 31843201

[60] Yue X Trifari S Aijo T Tsagaratou A Pastor WA Zepeda-Martinez JA. Control of Foxp3 stability through modulation of TET activity. J Exp Med (2016) 213(3):377–97. doi: 10.1084/jem.20151438 PMC4813667 26903244

[61] Garg G Nikolouli E Hardtke-Wolenski M Toker A Ohkura N Beckstette M. Unique properties of thymic antigen-presenting cells promote epigenetic imprinting of alloantigen-specific regulatory T cells. Oncotarget (2017) 8(22):35542–57. doi: 10.18632/oncotarget.16221 PMC5482597 28415767

[62] Sasidharan Nair V Song MH Oh KI. Vitamin c facilitates demethylation of the Foxp3 enhancer in a tet-dependent manner. J Immunol (2016) 196(5):2119–31. doi: 10.4049/jimmunol.1502352 26826239

[63] Nair VS Oh KI. Down-regulation of Tet2 prevents TSDR demethylation in IL2 deficient regulatory T cells. Biochem Biophys Res Commun (2014) 450(1):918–24. doi: 10.1016/j.bbrc.2014.06.110 24984151

[64] Chorro L Suzuki M Chin SS Williams TM Snapp EL Odagiu L. Interleukin 2 modulates thymic-derived regulatory T cell epigenetic landscape. Nat Commun (2018) 9(1):5368. doi: 10.1038/s41467-018-07806-6 30560927 PMC6299086

[65] Kitagawa Y Ohkura N Kidani Y Vandenbon A Hirota K Kawakami R. Guidance of regulatory T cell development by Satb1-dependent super-enhancer establishment. Nat Immunol (2017) 18(2):173–83. doi: 10.1038/ni.3646 PMC5582804 27992401

[66] Thiault N Darrigues J Adoue V Gros M Binet B Perals C. Peripheral regulatory T lymphocytes recirculating to the thymus suppress the development of their precursors. Nat Immunol (2015) 16(6):628–34. doi: 10.1038/ni.3150 25939024

[67] Nikolouli E Elfaki Y Herppich S Schelmbauer C Delacher M Falk C. Recirculating IL-1R2(+) tregs fine-tune intrathymic treg development under inflammatory conditions. Cell Mol Immunol (2021) 18(1):182–93. doi: 10.1038/s41423-019-0352-8 PMC7853075 31988493

[68] Owen DL La Rue RS Munro SA Farrar MA. Tracking regulatory T cell development in the thymus using single-cell RNA Sequencing/TCR sequencing. J Immunol (2022) 209(7):1300–13. doi: 10.4049/jimmunol.2200089 PMC9529998 36038290

[69] Huehn J Siegmund K Lehmann JC Siewert C Haubold U Feuerer M. Developmental stage, phenotype, and migration distinguish naive- and effector/memory-like CD4+ regulatory T cells. J Exp Med (2004) 199(3):303–13. doi: 10.1084/jem.20031562 PMC2211798 14757740

[70] Siewert C Menning A Dudda J Siegmund K Lauer U Floess S. Induction of organ-selective CD4+ regulatory T cell homing. Eur J Immunol (2007) 37(4):978–89. doi: 10.1002/eji.200636575 17345581

[71] Li C DiSpirito JR Zemmour D Spallanzani RG Kuswanto W Benoist C. TCR transgenic mice reveal stepwise, multi-site acquisition of the distinctive fat-treg phenotype. Cell (2018) 174(2):285–99.e12. doi: 10.1016/j.cell.2018.05.004 29887374 PMC6046274

[72] Li C Munoz-Rojas AR Wang G Mann AO Benoist C Mathis D. PPARgamma marks splenic precursors of multiple nonlymphoid-tissue treg compartments. Proc Natl Acad Sci USA (2021) 118(13):e2025197118. doi: 10.1073/pnas.2025197118 33753509 PMC8020779

[73] Miragaia RJ Gomes T Chomka A Jardine L Riedel A Hegazy AN. Single-cell transcriptomics of regulatory T cells reveals trajectories of tissue adaptation. Immunity (2019) 50(2):493–504.e7. doi: 10.1016/j.immuni.2019.01.001 30737144 PMC6382439

[74] Delacher M Imbusch CD Hotz-Wagenblatt A Mallm JP Bauer K Simon M. Precursors for nonlymphoid-tissue treg cells reside in secondary lymphoid organs and are programmed by the transcription factor BATF. Immunity (2020) 52(2):295–312.e11. doi: 10.1016/j.immuni.2019.12.002 31924477 PMC7026712

[75] DiSpirito JR Zemmour D Ramanan D Cho J Zilionis R Klein AM. Molecular diversification of regulatory T cells in nonlymphoid tissues. Sci Immunol (2018) 3(27):eaat5861. doi: 10.1126/sciimmunol.aat5861 30217811 PMC6219455

[76] Delacher M Imbusch CD Weichenhan D Breiling A Hotz-Wagenblatt A Trager U. Genome-wide DNA-methylation landscape defines specialization of regulatory T cells in tissues. Nat Immunol (2017) 18(10):1160–72. doi: 10.1038/ni.3799 PMC5912503 28783152

[77] Delacher M Schmidl C Herzig Y Breloer M Hartmann W Brunk F. Rbpj expression in regulatory T cells is critical for restraining TH2 responses. Nat Commun (2019) 10(1):1621. doi: 10.1038/s41467-019-09276-w 30962454 PMC6453958

[78] Garg G Muschaweckh A Moreno H Vasanthakumar A Floess S Lepennetier G. Blimp1 prevents methylation of Foxp3 and loss of regulatory T cell identity at sites of inflammation. Cell Rep (2019) 26(7):1854–68.e5. doi: 10.1016/j.celrep.2019.01.070 30759395 PMC6389594

[79] Schmidl C Delacher M Huehn J Feuerer M. Epigenetic mechanisms regulating T-cell responses. J Allergy Clin Immunol (2018) 142(3):728–43. doi: 10.1016/j.jaci.2018.07.014 30195378

[80] Baron U Floess S Wieczorek G Baumann K Grutzkau A Dong J. DNA Demethylation in the human FOXP3 locus discriminates regulatory T cells from activated FOXP3(+) conventional T cells. Eur J Immunol (2007) 37(9):2378–89. doi: 10.1002/eji.200737594 17694575

[81] Ohkura N Yasumizu Y Kitagawa Y Tanaka A Nakamura Y Motooka D. Regulatory T cell-specific epigenomic region variants are a key determinant of susceptibility to common autoimmune diseases. Immunity (2020) 52(6):1119–32.e4. doi: 10.1016/j.immuni.2020.04.006 32362325

[82] Delacher M Simon M Sanderink L Hotz-Wagenblatt A Wuttke M Schambeck K. Single-cell chromatin accessibility landscape identifies tissue repair program in human regulatory T cells. Immunity (2021) 54(4):702–20.e17. doi: 10.1016/j.immuni.2021.03.007 33789089 PMC8050210

[83] Itahashi K Irie T Yuda J Kumagai S Tanegashima T Lin YT. BATF epigenetically and transcriptionally controls the activation program of regulatory T cells in human tumors. Sci Immunol (2022) 7(76):eabk0957. doi: 10.1126/sciimmunol.abk0957 36206353

[84] Rudra D deRoos P Chaudhry A Niec RE Arvey A Samstein RM. Transcription factor Foxp3 and its protein partners form a complex regulatory network. Nat Immunol (2012) 13(10):1010–9. doi: 10.1038/ni.2402 PMC3448012 22922362

[85] Yang S Fujikado N Kolodin D Benoist C Mathis D. Immune tolerance. regulatory T cells generated early in life play a distinct role in maintaining self-tolerance. Science (2015) 348(6234):589–94. doi: 10.1126/science.aaa7017 PMC4710357 25791085

[86] Yang J Zou M Chu X Floess S Li Y Delacher M. Inflammatory perturbations in early life long-lastingly shape the transcriptome and TCR repertoire of the first wave of regulatory T cells. Front Immunol (2022) 13:991671. doi: 10.3389/fimmu.2022.991671 36119090 PMC9471859

[87] Hefazi M Bolivar-Wagers S Blazar BR. Regulatory T cell therapy of graft-versus-Host disease: Advances and challenges. Int J Mol Sci (2021) 22(18):9676. doi: 10.3390/ijms22189676 34575843 PMC8469916

[88] Juneja T Kazmi M Mellace M Saidi RF. Utilization of treg cells in solid organ transplantation. Front Immunol (2022) 13:746889. doi: 10.3389/fimmu.2022.746889 35185868 PMC8854209

